# Supplementary material for: Complexome profiling on the Chlamydomonas lpa2 mutant reveals insights into PSII biogenesis and new PSII associated proteins
Source: J Exp Bot. 2021 Aug 26;73(1):245–62. doi: 10.1093/jxb/erab390 (PMC8730698; doi:10.1093/jxb/erab390)
Supplement: erab390_suppl_Supplementary_Dataset_S1 [file erab390_suppl_supplementary_dataset_s1.zip › Supplemental Dataset 1 - Excel List and all profiles/plots/UAP56_Cre17.g719000.html]

Trivial name: UAP56  
  
Euclidean distance: 4445.07  
Mean Intensity (WT): 283.83  
Mean Intensity (Mut): 161.55  
Distance: 15.66  
  
MapMan: protein.synthesis.initiation;DNA.synthesis/chromatin structure;RNA.processing.RNA helicase  
  
p value of intensity sums Welch test: 0.5448
